# Overexpression of nuclear transport factor 2 may protect against diabetic retinopathy

**Published:** 2009-04-27

**Authors:** Bin Li, Hai-Qing Zhang, Yu Shi, Yun-Bing Min, Shao-Fen Lin, Kai-Li Wu, Jie Hu, Shi-Bo Tang

**Affiliations:** 1 Key Laboratory of Ophthalmology of the Ministry of Education and Zhong-Shan Ophthalmic Center, Sun Yet-Sun University, Guangzhou City, People’s Republic of China; 2 Department of Ophthalmology, Tongji Hospital, Tongji Medical College, Huazhong University of Science and Technology, Wuhan, People’s Republic of China; 3 Department of Ophthalmology, First Affiliated Hospital of Gannan Medical College, Gan Zhou, Jiangxi, People’s Republic of China; 4 Tungshan Area, People’s Hospital, Sun Yet-Sun University, Guangzhou City, People’s Republic of China

## Abstract

**Purpose:**

We performed human, animal, and in vitro studies to examine the potential role of nuclear transport factor 2 (NTF2) in conferring resistance to diabetic retinopathy (DR).

**Methods:**

Blood NTF2 levels were assessed in two groups of patients with type 2 diabetes mellitus. Group P patients had a history of proliferative DR (PDR), while group N patients did not. The retinal vasculature was examined in diabetic rats three months after they received an intravitreal injection of a recombinant adeno-associated virus (rAAV) vector overexpressing NTF2 (rAAV2-NTF2). Control rats were treated with rAAV2 only. Rat retinal capillary endothelial cells (RRCECs) were infected with rAAV2-NTF2, or with a vector expressing siRNA targeted against NTF2, to assess the effects of overexpression and inhibition of NTF2 on vascular endothelial growth factor (VEGF) expression (mRNA and protein).

**Results:**

There was a strong trend for patients with DR to have lower blood NTF2 levels compared to those who did not have DR (0.10±0.01 versus 0.20±0.08, p=0.079). There was significantly less retinal blood vessel leakage in diabetic rats infected with rAAV2-NTF2 compared to controls (16.5±2.9 versus 24.7±7.3, p=0.039). These rats exhibited normal retinal vasculature and blood-retinal barrier function. VEGF expression was inhibited by NTF2 overexpression and stimulated by NTF2 inhibition, (protein [0.41±0.05 versus 0.23±0.06] and mRNA [0.37±0.04 versus 0.23±0.06] p<0.01 for all).

**Conclusions:**

These finding suggest that NTF2 is a potential mediator of retinal vasculature integrity. NTF2 may act by altering VEGF expression, thereby influencing the development of DR in patients with diabetes mellitus.

## Introduction

Diabetic retinopathy (DR) and consequent vision loss or impairment is a common complication of type 2 diabetes mellitus (DM). To date, no effective therapy exists to treat this associated vision loss. Lack of glucose control and the chronic nature of the disease are key risk factors, but DR also contains a hereditary component. The aldose reductase (ALR2) gene (allele z-4) has been linked to the occurrence of DR [1]. Wang and colleagues [2] found that Chinese patients with type 2 DM exhibited phenotypic differences in terms of risk factors for DR, and that ALR2 was associated with microvascular complications. A study of Mexican Americans with DM revealed that retinopathy was linked to two chromosomes [3], while Suzuki et al. [4] reported that apolipoprotein was associated with the occurrence of DR. Other studies indicate that vascular endothelial growth factor (VEGF), genetic polymorphisms, and mitochondrial rRNA genetic polymorphisms may contribute to DR pathology [5,6].

The onset of DR among DM patients is variable. Some patients acquire DR as soon as they develop DM, while others either do not exhibit symptoms, or have very mild symptoms even many years after the onset of DM [7]. An epidemiologic study of American patients with a 20-year history of DM revealed that 80% had developed DR [8]. Evidently there is a distinct cohort of individuals with DM who are somehow “immune” to the onset of DR. These findings suggest the existence of a genetic polymorphism that protects retinal blood vessels from the damage associated with DM. Given this and the knowledge that no current medication can effectively control the occurrence and development of DR, it would seem pertinent to search for and try to identify genes that may be involved in conferring resistance to DR.

Nuclear transport factor 2 (NTF2) is a small guanosine 5′-diphosphate (GDP) Ran binding protein found in all human cell types. It is involved in regulating multiple processes, including cell cycle, immunoreactions, and apoptosis [9-11]. The main function of NTF2 is to facilitate transport of certain proteins into the nucleus via interaction with nucleoporin FxFG [12,13]. NTF2 also works as a GDP-dissociation inhibitor to mediate the GDP-Ran gradient, which is also involved nucleocytoplasmic transport [14-16]. The importance of NTF2 is illustrated by the findings that deletion of this gene results in lethality [17]. With regards to the eye, it has been reported that partial deletion or mutation of the NTF2 gene may cause heteroplasia in the eyes of *Drosophila* [17]. It has been further suggested that partial loss of NTF2 function might alter the nuclear import of Ran proteins during the immune response and that the loss of functional alleles may be associated with the strong eye phenotype [18].

In this study,we obtained blood samples from patients who suffered DM, but not proliferative DR (PDR), and from patients who suffered DM and PDR. RNA was extracted from these samples and subjected to microarray analysis to search for genes differentially expressed between the two patient groups and hence perhaps associated with DR. One of these was NTF2 (Table 1).

**Table 1:** Summary of significant differential gene expression as determined by microarray analysis.

**Gene**	**Description**	**p-value**
213726_x_at	nf66f09.s1 NCI_CGAP_Co3 Homo sapiens cDNA clone IMAGE:924905 3′ similar to gb:X79535 tubulin beta-2 chain (human); mRNA sequence.	0.00988
209458_x_at	hemoglobin, alpha 2	0.00985
206208_at	carbonic anhydrase IV	0.00981
1558459_s_at	MRNA; cDNA DKFZp686D21117 (from clone DKFZp686D21117)	0.00923
201230_s_at	ariadne homolog 2 (Drosophila)	0.00895
226975_at	hypothetical protein FLJ25070	0.00895
1553588_at	NADH dehydrogenase, subunit 3 (complex I); go_component: mitochondrial inner membrane [goid 0005743] [evidence P] [pmid 9878551]; go_component: respiratory chain complex I (sensu Eukarya) [goid 0005747] [evidence NAS]; go_component: mitochondrion [goid 0005739] [evidence IEA]; go_function: NADH dehydrogenase (ubiquinone) activity [goid 0008137] [evidence NAS] [pmid 9878551]; go_function: oxidoreductase activity [goid 0016491] [evidence IEA]; go_process: mitochondrial electron transport, NADH to ubiquinone [goid 0006120] [evidence NAS] [pmid 9878551]; Homo sapiens NADH dehydrogenase 3 (MTND3), mRNA.	0.00892
226434_at	hypothetical protein MGC22793	0.0084
201788_at	DEAD (Asp-Glu-Ala-Asp) box polypeptide 42	0.00824
223440_at	lin-10 protein homolog	0.00802
243954_at	Hypothetical protein LOC285286 (LOC285286), mRNA	0.00781
205844_at	vanin 1	0.00673
228460_at	zinc finger protein 319	0.00671
236012_at	proteasome (prosome, macropain) inhibitor subunit 1 (PI31)	0.00663
233690_at	CDNA: FLJ23090 fis, clone LNG07119	0.00576
202397_at	nuclear transport factor 2	0.00542
224776_at	putative lysophosphatidic acid acyltransferase	0.00529
201378_s_at	NICE-4 protein	0.00515
223365_at	DEAH (Asp-Glu-Ala-His) box polypeptide 37	0.00469
217232_x_at	Homo sapiens mutant beta-globin (HBB) gene, complete cds	0.00402
211745_x_at	hemoglobin, alpha 2	0.00385
219226_at	CDC2-related protein kinase 7	0.00286
225819_at	transforming growth factor beta regulator 1	0.000514

The aim of the current study was to determine if NTF2 plays a role in conferring resistance to DR. A series of studies were performed. NTF2 gene expression was determined in a cohort of DM patients divided into those with PDR and those without. In vivo studies with diabetic rats and in vitro studies using rat retinal capillary endothelial cells (RRCECs)were performed to assess the effects of overexpression and inhibition of NTF2 expression on retinal vasculature and inhibition of retinal damage.

## Methods

### Human study

Patients were classified into two groups: those without PDR (group N) and those with PDR (group P). Group N patients had a 20 to 25 year history of DM without PDR as confirmed by retinal photography and fluorescein angiography. Group P patients had a 20 to 25 year history of type 2 DM with active PDR in both eyes as confirmed by retinal photography. 59 patients in both groups were of similar age (average age 67.3 years old), weight, ethnicity (Han Chinese), equal gender distribution and resided in or around Guangzhou City, China. Living standards of patients in both groups were similar. All patients had been on insulin therapy for approximately two years. Glucose levels before and after meals were strictly controlled in Group N but not in Group P. The diagnosis of PDR was made in accordance with international standards. Patients with malignant tumors, other hereditary diseases, severe infectious diseases (i.e., tuberculosis, hepatitis B) by biochemical examination and asked about the disease history, and hypertension or unstable critical condition were excluded. A total of 22 and 37 patients met the inclusion criteria for groups N and P over a period of 14 months. Blood samples (2.5 ml) were obtained from 20 patients (we selected) in each group for microarray analysis. Thereafter, six blood samples from each group of patients were selected, along with samples from a cohort of healthy volunteers controls (the NO group, 6 patients were recruited, they live in Guangzhou China, and average age of 68.5 years, the blood samples was frozen in −20 °C), for evaluation of NTF2 gene expression.

This research was approved by the ethics committee of Zhongshan Ophthalmic Center, Sun Yet-Sun University. Each patient signed a consent statement before entering the study.

### Quantitative real time PCR evaluation of *NTF2* gene expression

Total RNA was isolated from patient blood samples using Trizol reagent (Gibco, Los Angeles, CA) and purified by Qiagen RNeasy Mini Kit (Qiagen, Venlo, The Netherlands). Synthesis of cDNA was performed using a Super-Script II cDNA synthesis Kit (Invitrogen Inc., Carlsbad, CA). Real-time PCR (RT–PCR) was performed using a Hotstar Taq polymerase kit (Qiagen) with SYBR Green technology (Applied Biosystems Inc., Foster City, CA) according to the manufacturer’s instructions. Relative gene expression was determined. The primers used to detect 384 bp NTF2 are described in Table 2.

**Table 2:** Quantitative real time PCR evaluation of *NTF2* gene expression.

**Gene**	**Primer (5′-3′)**	**PCR annealing temp (°C)**
*NTF2*	AGCTTAAGGCGGATGAAGACC	58
	GAGGAGGAAACAGCGTGAGTG	
*β-actin*	CTTTTAGGATGGCAAGGGACT	58
	TGGAACGGTGAAGGTGACA	

### Construction and packaging of rAAV2-NTF-2

The coding sequence for *NTF2* was obtained from the Sprague-Dawley (SD) rat cDNA bank and was extended using Golden Taq (Tiangen Biotech, Beijing, China). The method for construction and packaging rAAV2-NTF-2 has been described previously [19]: The oligonucleotide upper primer are described in Table 3, polymerase chain reaction (PCR) amplification was performed in a final volume of 50 μl. The following temperature conditions were used for the reactions: 3 min initial denaturation at 94 °C, then 32 cycles for 30 s denaturation at 94 °C; 30 s annealing at 56 °C, synthesis for 1 min at 72 °C. The amplified product was subcloned directly into vector pGEM-T. Gene sequence was verified by dideoxynucleotide sequencing. Digestion of the recombinant plasmid pGEM-T-NTF2 with EcoRI acquired the cDNA of NTF2 extracellular domain which was subcloned into the EcoRI site of the gene therapy vector pSNAV-2. Recombinant plasmid was examined by restriction enzyme digestion and named pSNAV- 2/NTF2.

**Table 3:** Cloning and sequencing of the rat *NTF2* gene.

**Gene**	**Primer (5′-3′)**	**PCR annealing temp (°C)**
*NTF2*	GGAATTCATGGGAGACAAGCCAATTG	56
	GGAATTCTCAGCCGAAGTTGTGC	

Transfection of baby hamster kidney (BHK-21) cells with cells with pSNAV-NTF-2 was performed in six-well plates using a Lipofectamine 2000 kit (Invitrogen). These cells, named BHK/NTF-2, were then transferred and cultured in a 110 mm×480 mm flask (Wheaton Inc., Wheaton, IL), then infected with HSV1-rc/ΔUL2 (Benyuan Zhengyang Inc., Beijing, China; MOI=0.1) when the cell number reached 8×10^8^. The cells were then divided into 250 ml Fernbach culture flasks for further purification after 48 h culture.

The rAAV2-NTF-2 virus was purified and identified using RT–PCR as described previously [19]. The titer of rAAV2-NTF-2 (virus genome/ml [vg/ml]) was detected using in situ hybridization with a digoxin-labeled cytomegalovirus probe. The final titer was 1×10^12^ virus genomes/ml.

### In vivo study: overexpression of the *NTF2* gene in the rat retina

Obtained from the animal center of Huazhong University of Science and Technology were 60, two-month-old male Sprague Dawley rats, weighing between 150 and 200 g. Rats maintained in an cleaning environment for two weeks before experimentation. Rats were randomly divided into two groups, A and B, and containing 30 rats per group. Each rat in Group A received an intravitreal injection of purified rAAV2-NTF2 in either the left or right eye (determined randomly). Rats in Group B were injected with rAAV2 only. Diabetes animal model were Constructed after rAAV2 treatment one month: after fasting for 12 h [20], the rats were injected with a single dose of streptozotocin (STZ; 65 mg/kg intraperitoneal injection in 0.01 M citrate buffer with a pH of 4.5). Nondiabetic control mice received citrate buffer only. Fasting plasma glucose was examined after 3 days of STZ injection, and diabetes was confirmed by fasting plasma glucose value of 16.7 mmol/l or higher using Touch™ Glucometer (Boehringer Mannheim Diagnostics, Indianapolis, IN). Retinal integrity was assessed two months later.

#### Assessment of retinal blood vessel leakage

Retinal vascular permeability was determined by assessing fluorescein isothiocyannate-dextran (FITC- dextran) [21-23] and Evans Blue (EB) retinal leakage [24]. Rats were anesthetized by intraperitoneal injection of 50 mg/kg ketamine and 20 mg/kg chlorpromazine. Next, a 100 mg/kg solution of FITC-dextran solution (Sigma, St Louis, MO) or 45 mg/kg of 3% EB (Sigma) was injected via the tail vein. The chest was opened after 2 h and an infusion tube connected with a 14G blunt needle was inserted into the left ventricle. The right atrial appendage was cut open, and the heart was infused with fluid contained 0.05 M 1% paraformaldehyde citrate buffer, pH 3.5, at 250 ml/kg bodyweight. Infusion height was 160 cm, resulting in an equivalent infusion pressure of 120 mmHg. The infusion was maintained for 2 min to remove any remnant dye in the blood vessels. The eyes of rat were then removed, and the retina was dissected and mounted to determine retinal blood vessel leakage (FITC-dextran leakage) by microscopy.

To assess EB leakage, we dried dissected retinas under vacuum (45 °C for 5 h). The retinal dry weight was recorded. The difference in retinal weight between diabetic rats injected with rAAV2-NTF2 and controls was compared. Next, 120 μl formamide was added and the solution placed in a 70 °C water bath for 18 h. The sample was then centrifuged at 7,280 xg for 30 min to separate the dye from the protein. The absorbance of the 50 μl filtered fluid was detected at wavelengths of 620 nm and 740 nm using a Beckman DU-640 spectrophotometer (Beckman Coulter, Fullerton, CA). The net absorbance was calculated by subtracting the absorbance at 740 nm from that at 620 nm. The concentration of dye was calculated from a standard curve (determined for each measurement) of EB in formamide. Samples were analyzed in triplicate and the mean calculated. The dry weight of the retina (mg) was used to standardize dye content and values are presented in ng/mg. The formula was as follows:

EB content in the retina (ng/mg)=[EB concentration in formamide (ng/μl)×120 (μl)]/dry weight of the retina (mg)

### In vitro studies: inhibition of *NTF2* expression using siRNA

In vitro cultured RRCECs were prepared as previously described [25]. Briefly, rat eyes were cut circumferentially 1.5 mm posterior to the limbus, and the retinas were harvested and homogenized by two gentle up-and-down strokes in a 15 ml homogenizer (Dounce; Bellco Glass Co., Vineland, NJ). The homogenate was filtered. The remaining retentate was digested in 0.066% collagenase for 45 min at 37 °C. The homogenate was centrifuged (1,000x g for 10 min), and the pellet was resuspended in human serum-free endothelial-basal growth medium Invitrogen-Gibco, Grand Island, NY), supplemented with 20% fetal bovine serum, 50 U/ml endothelial cell growth factor (Sigma-Aldrich). Cells were cultured in fibronectin-coated dishes and incubated at 37 °C in a humidified atmosphere containing 5% CO_2_. Cultured RRCECs were transfected with rAAV2-NTF2, or rAAV2-GFP. Cells were plated in 24 well culture plates, grown overnight to 70%–80% confluence. Cells were then washed twice with serum-free human endothelial-serum-free medium basal growth medium. Next, 1×10^9^ vg/ml rAAV2-NTF2 was added to each well in the experimental group while 1×10^9^ vg/ml rAAV2 only was added to control wells. rAAV2-GFP was added to six separate wells to monitor infection efficiency (subsequently found to approximate 93%).

A vector containing siRNA for NTF2 was constructed by cloning the double stranded sequence into the RNAi-Ready pSIREN-RetroQ ZsGreen vector (pSIREN vector; BD Biosciences, Franklin Lakes, NJ) to yield the pSIREN-NTF2 siRNA vector. The *NTF2* sequences (obtained from Qiagen) were described in Table 4. RRCECs were infected with pSIREN-NTF2 siRNA or pSIREN vectors using Lipofectamine. Real time PCR and western blot analyses were performed to examine the RNA and protein expression levels of *NTF2* and *VEGF* two days after infection.

**Table 4:** The RNAi *NTF2* sequence.

**Primer**	**Sequence**
Sense	GATCCAAGCCAATTTGGGAGCAGATTTTCAAGAGAAATCTGCTCCCAAATTGGCTTTTTTTTACGCGTG
Antisense	ATTCACGCGTAAAAAAAAGCCAATTTGGGAGCAGATTTCTCTTGAAAATCTGCTCCCAAATTGGCTTG

#### Quantitative real time PCR and western blot evaluation of NTF2 and VEGF gene and protein expression

Total RNA was isolated from RRCECs using Trizol reagent (Gibco, Los Angeles, CA) and purified by Qiagen RNeasy Mini Kit (Qiagen, Venlo, The Netherlands). Synthesis of cDNA was performed using a Super-Script II cDNA synthesis Kit (Invitrogen Inc.). Real-time PCR was performed using a Hotstar Taq polymerase kit (Qiagen) with SYBR Green technology (Applied Biosystems Inc., Foster City, CA) according to the manufacturer’s instructions. Relative gene expression was determined. The primers were described in Table 5. Total protein was extracted from RRCECs using bicinchoninic acid. Isolated protein was mixed with loading buffer, denatured for 6 min at 60 °C, cooled, centrifuged for 5 min, and then separated by sodium-dodecyl sulfate PAGE (SDS–PAGE). Antibodies directed toward NTF2 and VEGF (Santa Cruz Biotechnology Inc., Santa Cruz, CA) were used to probe the proteins. A secondary antibody goat anti-mouseIgG-HRP (1:1,000 dilution, Santa Cruz Biotechnology Inc., Santa Cruz, CA) was applied, and the signal was revealed by chemiluminescence. The polyvinylidene fluoride membranes were reused to detect β-actin (internal control) by incubating with a mouse anti-human actin antibody (Gene Co, Hong Kong, China). The bands detected were analyzed by automatic image analysis, and the integrated optical density (OPTDI) of each protein band was normalized to the OPTDI value of the corresponding β-actin band from the same sample.

**Table 5:** Quantitative real time PCR evaluation of *NTF2* AND *VEGF* expression.

**Gene**	**Primer (5′-3′)**	**PCR annealing temp (°C)**
*NTF2*	AAAGAACATCAATGACGCTTGG	57
	GGAGCATCTGGAGGAGATAGGA	
*β-actin*	CTGGGTATGGAATCCTGTGG	58
	TCATCGTACTCCTGCTTGCTG	
*VEGF*	TCTTCAAGCCGTCCTGTGTG	58
	ACAGTGAACGCTCCAGGATTTA	

### Statistical analysis

Normally distributed data were compared by independent two samples *t*-test or one-way ANOVA where appropriate. When a significant difference was detected between groups differences were detected, multiple comparisons of means were performed using the Bonferroni procedure, with type-I error rate at a maximum of 0.017(0.05/3) adjustment. Statistical analyses were performed using SPSS 15.0 statistics software (SPSS Inc., Chicago, IL), and differences were considered significant when p<0.05. Data are presented as the mean±standard deviation (SD).

## Results

### *NTF2* expression in patients

**Figure 1:**
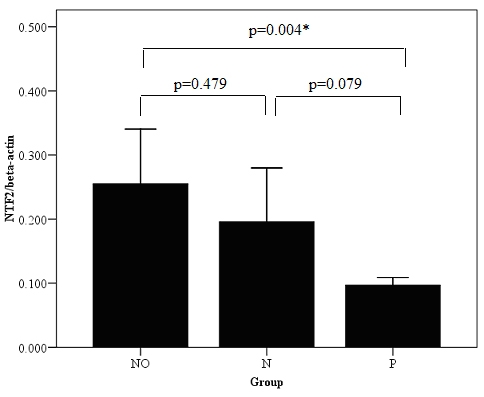
*NTF2* expression was significantly lower in patients with PDR. Expression of *NTF2* mRNA as determined by real-time PCR in patients with type 2 diabetes mellitus (DM) and proliferative diabetic retinopathy (PDR, group P), DM without PDR (group N) and healthy volunteers (group NO). Asterisk indicates significance between groups difference (p<0.05).

There was a significant overall difference in *NTF2* gene expression among the groups (NO, N, and P). *NTF2* expression was lower in group N patients compared to NO (control), however this difference was insignificant (0.20±0.08 versus 0.26±0.09, p=0.479; Figure 1). *NTF2* expression was significantly higher in normal compared to group P patients (0.26±0.09 versus 0.10±0.01; p=0.004). There was no significant difference in *NTF2* expression between group N and P patients.

### Effect of *NTF2* overexpression on retinal blood vessel leakage in rats with DM

**Figure 2:**
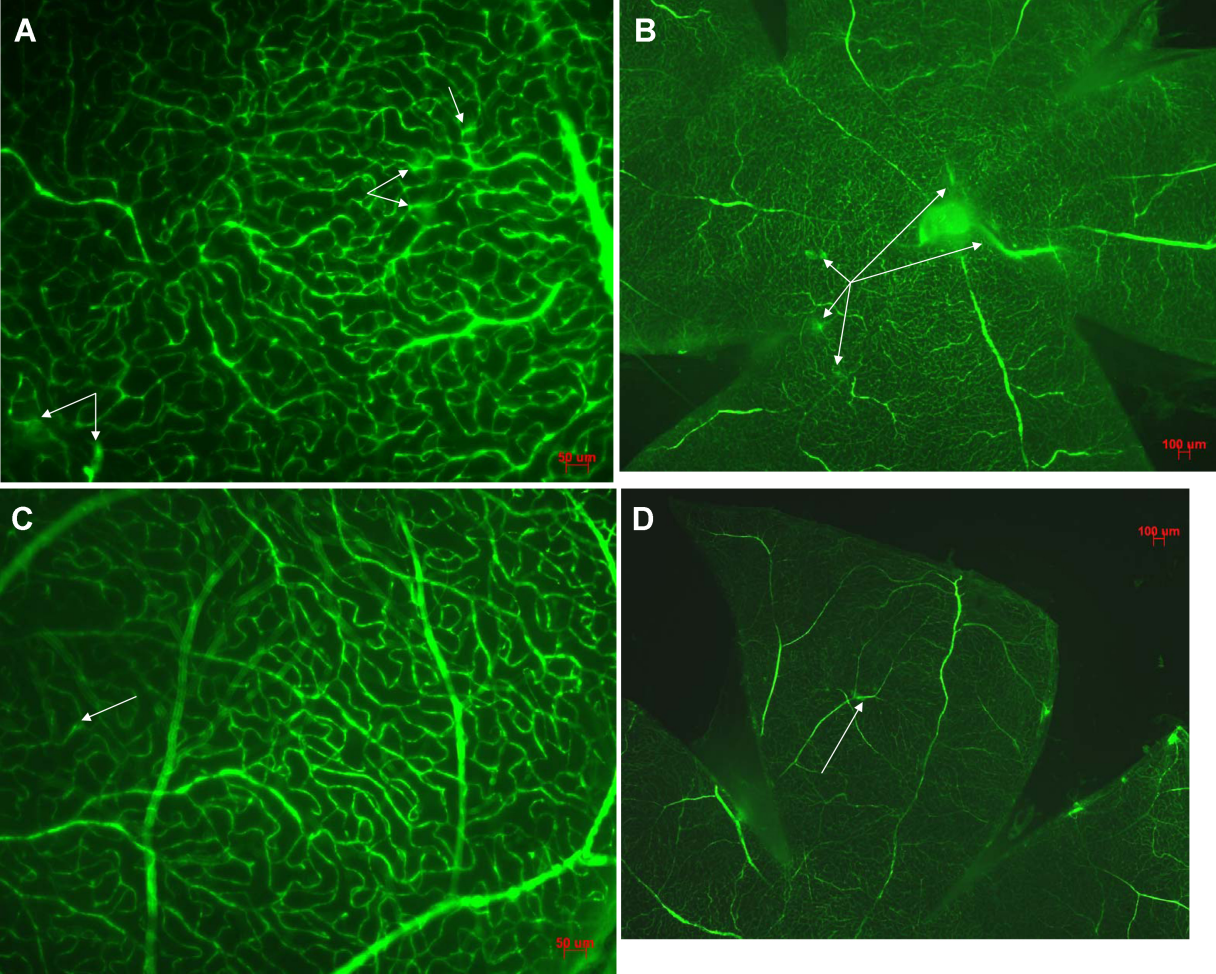
Retinal sections from diabetic rats infused with fluorescein isothiocyanate. Representative images from rAAV2-infected rats was shown on **A** and **B.** Representative images from rAAV2-NTF2-infected rats were shown on **C** and **D.** Decreased blood vessel leakage was apparent in retinas from group B rats (NTF2 overexpression). Scale bar=50 μm for **A** and **C**, 100 μm for **B** and **D**.

The FITC-dextran blood vessel leakage area was significantly decreased in rats treated with rAAV2-NTF2 compared to in control rats (11.3±1.5% versus 23.7±2.8%; p<0.05). Representative images demonstrating retinal leakage of FITC dye can be seen in Figure 2.

There was significantly less EB content in retinas from rAAV2-NTF2 injected as compared to control rats (16.5±2.9 ng/mg retinal dry weight versus 24.7±7.3 ng/mg retinal dry weight; p=0.039).

### Effects of *NTF2* overexpression and downregulation on *VEGF* expression in RRCECs

**Figure 3:**
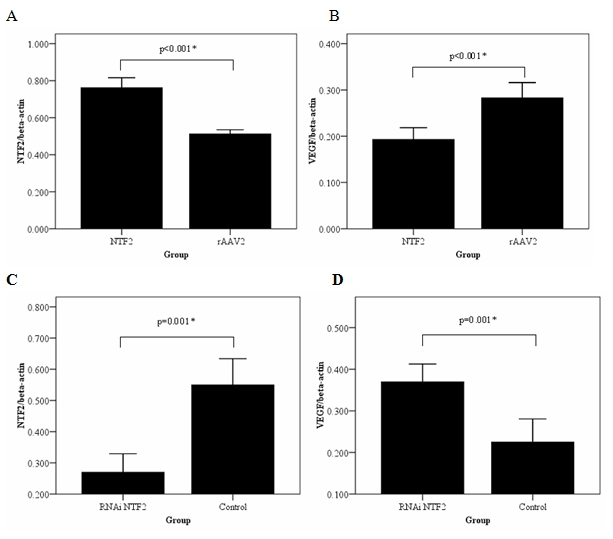
Effects of NTF2 levels on *NTF2* and *VEGF* mRNA expression. Overexpression of *NTF2* by transfection of rAAV2-NTF2 increased the expression level of NTF2 but reduced the expression of *VEGF* (**A, B**). Downregulation of *NTF2* by transfection with pSIREN-NTF2 siRNA reduced *NTF2* expression but increased *VEGF* expression (**C, D**). The experiments were performed in rat retinal capillary endothelial cells. Asterisk (*) indicates a significant difference between groups (p<0.05).

**Figure 4:**
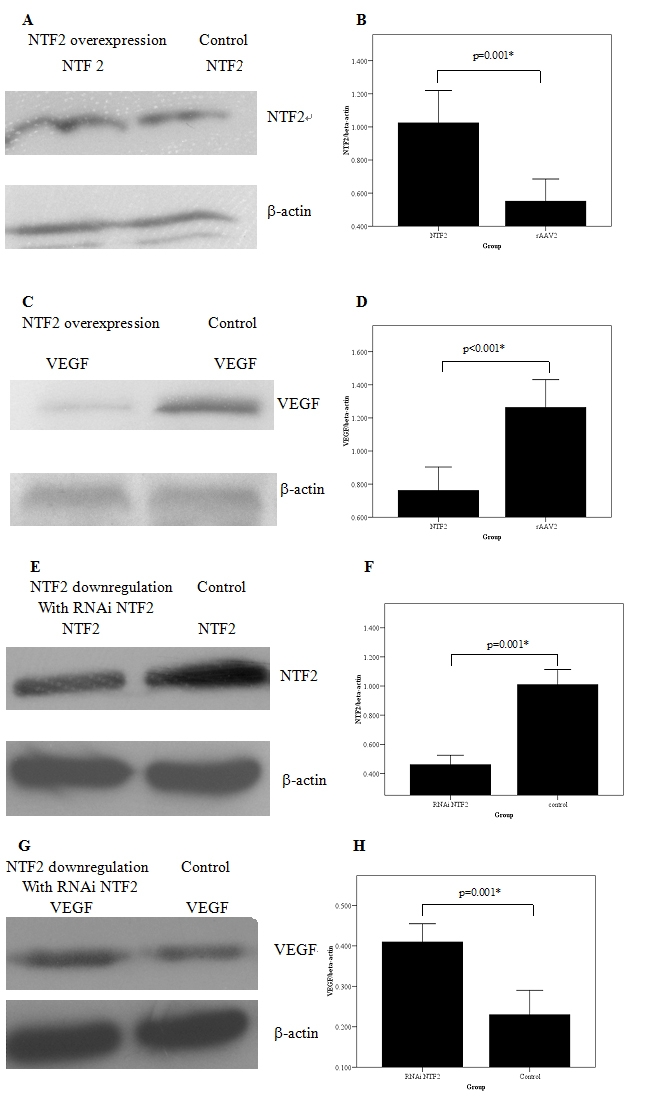
Effects of NTF2 expression on NTF2 and VEGF protein levels. Overexpression of NTF2 by transfection with rAAV2-NTF2 increased NTF2 protein expression but reduced VEGF protein level (**B, D**). Downregulation of NTF2 by transfection with pSIREN-NTF2 siRNA inhibited NTF2 protein expression but increased VEGF protein level (**F, H**). The experiments were performed in rat retinal capillary endothelial cells. Representative western blots were shown for the effects of NTF2 overexpression and downregulation on NTF2 (**A, E**) and VEGF (**C, G**) preotein expression. Asterisk (*) indicates a significant difference between the groups (p<0.05).

Real time PCR revealed that infection of RRCECs with rAAV2-NTF2 resulted in significant upregulation of *NTF2* mRNA (0.77±0.05 versus 0.51±0.02, p<0.01), In contrast, *VEGF* mRNA were significantly decreased in these cells (0.19±0.03 versus 0.28±0.03, p<0.01). *NTF2* mRNA were significantly decreased following transfection with pSIREN-NTF2 siRNA (0.27±0.06 versus 0.55±0.08; p<0.01). While *VEGF* mRNA were significantly increased in these compared to control cells (0.37±0.04 versus 0.23±0.06; p<0.01). Western blot analyses confirmed that Infection of RRCECs with rAAV2-NTF2 resulted in significant upregulation of NTF2 protein expression (1.03±0.20 versus 0.55±0.13,Figure 3A and Figure 4B; p<0.01). In contrast, VEGF protein levels (0.76±0.14 versus 1.26±0.17) were significantly decreased in these cells (Figure 3B and Figure 4D; p<0.01). NTF2 protein expression levels were significantly decreased following transfection with pSIREN-NTF2 siRNA (Figure 3C and 0.46±0.07 versus 1.01±0.10; Figure 4F; p<0.01). While VEGF protein levels were significantly increased in these compared to control cells (Figure 3D and 0.41±0.05 versus 0.23±0.06; Figure 4H; p<0.01)

## Discussion

The results of this study suggest that NTF2 may be involved in mediating DR in patients with DM. Several lines of evidence support this assertion. We found *NTF2* mRNA expression to be lower in DM patients with PDR (P group) compared to those without PDR. Even though the difference in humans was not statistically significant, we also found overexpression of *NTF2* in diabetic rats was associated with decreased indices of retinal damage. Findings from our in vitro studies of RRCECs, in which *NTF2* expression was upregulated or downregulated, indicated that NTF2 modulates VEGF expression. This is an important observation given that NTF2 may play a role in mediating the progression of DR and suggests the need for further studies, particularly in humans.

DR is among the most prevalent complications associated with DM. Hence, it is closely related to DM. Several studies have suggested that there is a genetic component to PDR [2,3,6,26]. It would appear that genes involved help maintain functionality of the retinal blood vessels and protects the surrounding tissue from DM-related damage.

At the beginning of study, we examined *NTF2* expression levels in patients, who had DM with or without PDR for 20–25 year. Potential confounding factors (e.g., age, gender, types of illness, and living conditions) were eliminated due to a strict selection and exclusion criteria of patients. Hence the differentiating factor between the 2 groups of patients was the presence or absence of PDR. We found that NTF2 levels in patients with PDR were significantly lower than in normal healthy individuals (NO) While there was no significant difference detected between the patient groups, there was a strong trend for patients with PDR to have lower NTF2 levels (p=0.079). This lack of a difference between these groups may be a reflection of the relatively small sample size. Nevertheless, this finding encouraged us to further examine the importance of NTF2 with regards to the development of DR.

Findings from a study by Minakhina and colleagues [17] have interesting parallels to our own. These researchers found that low *NTF2* gene expression resulted in abnormal eye growth in Drosophila. Higher *NTF2* gene expression was required to maintain normal eye growth. The number of compound eyes was significantly decreased in Drosophila with reduced as compared to normal *NTF2* expression. The decreased frequency of compound eyes in low *NTF2* expression Drosophila may have been due to dysplasia of blood vessels in the eye.

Given that lower *VEGF* expression is thought to be associated with DR resistance [27], it is plausible that NTF2 may offer protection from DR by depressing *VEGF* expression. Indeed our findings suggest that this may be the case (at least in part). We found that expression of NTF2 in RRCECs was inversely related to VEGF expression (both mRNA and protein). Precisely how NTF2 might influence VEGF expression is unclear. Further studies examining the effects of NTF2 on downstream inflammatory and vascular factors are warranted to clarify this point. Certainly, NTF2 may influence other mediators of DR aside from VEGF.

In summary, the findings from our study implicate NTF2 as a potential mediator of retinal vasculature protection and suggest that expression levels of this gene may dictate, at least to some extent, development of PDR in patients with DM. Our findings also suggest that NTF2 may exert such effects by altering VEGF expression. Further studies are warranted to examine potentials pathways through which NTF2 may be exerting such effects.

## References

[1] Warpeha KM, Chakravarthy U (2003). Molecular genetics of microvascular disease in diabetic retinopathy.. Eye.

[2] Wang Y, Ng MC, Lee SC, So WY, Tong PC, Cockram CS, Critchley JA, Chan JC (2003). Phenotypic heterogeneity and associations of two aldose reductase gene polymorphisms with nephropathy and retinopathy in type 2 diabetes.. Diabetes Care.

[3] Hallman DM, Boerwinkle E, Gonzalez VH, Klein BE, Klein R, Hanis CL (2007). A genome-wide linkage scan for diabetic retinopathy susceptibility genes in Mexican Americans with type 2 diabetes from Starr County, Texas.. Diabetes.

[4] Suzuki T, Oba K, Igari Y, Matsumura N, Inuzuka Y, Kigawa Y, Matsuura Y, Ajiro Y, Okazaki K, Nakano H (2002). Relation of apolipoprotein (a) phenotypes to diabetic retinopathy in elderly type 2 diabetes.. J Nippon Med Sch.

[5] Awata T, Inoue K, Kurihara S, Ohkubo T, Watanabe M, Inukai K, Inoue I, Katayama S (2002). A common polymorphism in the 5′-untranslated region of the VEGF gene is associated with diabetic retinopathy in type 2 diabetes.. Diabetes.

[6] Kuze M, Arima M, Saso M, Uji Y (1998). A familial case of proliferative diabetic retinopathy associated with a mutation in the mitochondrial gene.. Nippon Ganka Gakkai Zasshi.

[7] Cejkova P, Novota P, Cerna M, Kolostova K, Novakova D, Kucera P, Novak J, Andel M, Weber P, Zdarsky E (2008). HLA DRB1, DQB1, and insulin promoter VNTR polymorphisms: interactions and the association with adult-onset diabetes mellitus in Czech patients. Int J Immunogenet.

[8] Klein R, Klein BE, Moss SE, Davis MD, DeMets DL (1984). The Wisconsin epidemiologic study of diabetic retinopathy. III. Prevalence and risk of diabetic retinopathy when age at diagnosis is 30 or more years.. Arch Ophthalmol.

[9] Makhnevych T, Lusk CP, Anderson AM, Aitchison JD, Wozniak RW (2003). Cell cycle regulated transport controlled by alterations in the nuclear pore complex.. Cell.

[10] Bhattacharya A, Steward R (2002). The Drosophila homolog of NTF-2, the nuclear transport factor-2, is essential for immune response.. EMBO Rep.

[11] Ferrando-May E (2005). Nucleocytoplasmic transport in apoptosis.. Cell Death Differ.

[12] Morrison J, Yang JC, Stewart M, Neuhaus D (2003). Solution NMR study of the interaction between NTF2 and nucleoporin FxFG repeats.. J Mol Biol.

[13] Isgro TA, Schulten K (2007). Association of nuclear pore FG-repeat domains to NTF2 import and export complexes.. J Mol Biol.

[14] Ribbeck K, Lipowsky G, Kent HM, Stewart M, Gorlich D (1998). NTF2 mediates nuclear import of Ran.. EMBO J.

[15] Yamada M, Tachibana T, Imamoto N, Yoneda Y (1998). Nuclear transport factor p10/NTF2 functions as a Ran-GDP dissociation inhibitor (Ran-GDI).. Curr Biol.

[16] Paradise A, Levin MK, Korza G, Carson JH (2007). Significant proportions of nuclear transport proteins with reduced intracellular mobilities resolved by fluorescence correlation spectroscopy.. J Mol Biol.

[17] Minakhina S, Myers R, Druzhinina M, Steward R (2005). Crosstalk between the actin cytoskeleton and Ran-mediated nuclear transport.. BMC Cell Biol.

[18] Steggerda SM, Black BE, Paschal BM (2000). Monoclonal antibodies to NTF2 inhibit nuclear protein import by preventing nuclear translocation of the GTPase Ran.. Mol Biol Cell.

[19] Snyder RO, Xiao X, Samulski RJ. Production of recombinant adeno-associated viral vectors. In: Dracopoli N, editor. Current Protocols in Human Genetics. New York: John Wiley; 1996. p. 1-24. 10.1002/0471142905.hg1209s53 18428408

[20] Lopes de Faria JM, Silva KC, Boer PA, Cavalcanti TC, Rosales MA, Ferrari AL, Lopes de Faria JB (2008). Decrease in retinal progenitor cells is associated with early features of diabetic retinopathy in a model that combines diabetes and hypertension.. Mol Vis.

[21] D'Amato R, Wesolowski E, Smith LE (1993). Microscopic visualization of the retina by angiography with high-molecular-weight fluorescein-labeled dextrans in the mouse.. Microvasc Res.

[22] Paques M, Tadayoni R, Sercombe R, Laurent P, Genevois O, Gaudric A, Vicaut E (2003). Structural and hemodynamic analysis of the mouse retinal microcirculation.. Invest Ophthalmol Vis Sci.

[23] Scheppke L, Aguilar E, Gariano RF, Jacobson R, Hood J, Doukas J, Cao J, Noronha G, Yee S, Weis S, Martin MB, Soll R, Cheresh DA, Friedlander M (2008). Retinal vascular permeability suppression by topical application of a novel VEGFR2/Src kinase inhibitor in mice and rabbits.. J Clin Invest.

[24] Sima J, Ma J, Zhang SX, Guo J (2006). Study of the influence of angiostatin intravitreal injection on vascular leakage in retina and iris of the experimental diabetic rats.. Yan Ke Xue Bao.

[25] Li B, Tang SB, Hu J, Gao Y, Zhang G, Lin SF, Chen JH, Li BJ (2006). Protective effects of transcription factor HESR1 on retinal vasculature.. Microvasc Res.

[26] Hanis CL, Hallman D (2006). Genetics of diabetic retinopathy.. Curr Diab Rep.

[27] Smith A, Brownawell A, Macara IG (1998). Nuclear import of Ran is mediated by the transport factor NTF2.. Curr Biol.

